# Metabolic Imbalances and Bone Remodeling Agents in Adolescent Idiopathic Scoliosis: A Study in Postmenarcheal Girls

**DOI:** 10.3390/ijms241713286

**Published:** 2023-08-27

**Authors:** Anna Danielewicz, Magdalena Wójciak, Ireneusz Sowa, Monika Kusz, Joanna Wessely-Szponder, Sławomir Dresler, Michał Latalski

**Affiliations:** 1 Paediatric Orthopaedic Department, Medical University of Lublin, Gębali 6, 20-093 Lublin, Poland; michallatalski@umlub.pl; 2 Department of Analytical Chemistry, Medical University of Lublin, Chodźki 4a, 20-093 Lublin, Poland; magdalena.wojciak@umlub.pl (M.W.); i.sowa@umlub.pl (I.S.); dresler.slawomir@gmail.com (S.D.); 3 Department of Pediatric Nephrology, Childrens’ University Hospital in Lublin, Gębali 6, 20-093 Lublin, Poland; moniakusz@gmail.com; 4 Sub-Department of Pathophysiology, Department of Preclinical Veterinary Sciences, Faculty of Veterinary Medicine, University of Life Sciences, 20-033 Lublin, Poland; joanna.wessely@up.lublin.pl

**Keywords:** scoliosis, deformity, phosphate–calcium metabolism, FGF23, PINP, Klotho

## Abstract

The causes and mechanisms underlying adolescent idiopathic scoliosis (AIS) remain unclear, and the available information regarding metabolic imbalances in AIS is still insufficient. This investigation aimed to evaluate the concentrations of specific bone remodeling-related agents in postmenarcheal girls diagnosed with AIS. The study encompassed thirty-six scoliosis patients and eighteen age-matched healthy individuals assigned to the control group. The patients underwent clinical and radiological examinations to assess the degree of the spinal deformity, type of curvature, and skeletal maturity. Blood and urine samples were collected from all participants and serological markers were measured using an enzyme-linked immunosorbent assay. Our study results demonstrated that the balance of phosphate–calcium and parathormone levels seems normal in individuals with AIS. Furthermore, no statistically significant differences were observed in the content of Klotho protein, osteocalcin, osteoprotegerin, C-terminal telopeptide of type I collagen (CTX), sclerostin, and alkaline phosphatase. Nevertheless, the serum levels of vitamin D (25-OH-D) were lowered, while N-terminal propeptide of type I procollagen (PINP), and fibroblast growth factor-23 (FGF23) were increased in the AIS group, with *p*-values of 0.044, 0.001, and 0.022, respectively. This finding indicates the potential involvement of these factors in the progression of AIS, which necessitates further studies to uncover the fundamental mechanisms underlying idiopathic scoliosis.

## 1. Introduction

Adolescent idiopathic scoliosis (AIS) is a spinal deformity affecting approximately from 0.5% to 5% of children in the general population. Its primary feature is its spontaneous development during the child’s growth with a progressive tendency. Consequently, around 10% of patients require some form of treatment, and 0.1% necessitate surgery. Scoliosis is characterized by a lateral curvature of the spine with vertebral rotation, and typically, hypokyphosis of the thoracic spine. The main diagnostic criterion is a spinal curvature exceeding 10° of the Cobb angle observed on a plain anteroposterior X-ray image. This parameter is commonly used to quantify the magnitude of spinal deformity and is defined as the angle between the superior endplate of an upper vertebra and the inferior endplate of a lower vertebra. Scoliosis is labelled as idiopathic when no other underlying disease can be identified [1].

In mild spinal curves, treatment is based on rehabilitation with physical therapy. In moderate cases, spinal bracing is the standard method and the most widely used approach to correct spinal deformity and prevent the progression of the scoliosis [2,3,4]. Patients with a Cobb angle greater than 40 degrees often require surgical treatment, and various surgical systems may be applied to correct the spinal deformity [5,6,7,8]. The aim of surgery in the treatment of AIS is to improve cosmesis and function with low complication rates and minimal long-term implications.

The AIS etiology is still debated, but it is believed to have a multifactorial background. Several factors may contribute to the progression of the spinal curve, including genetic and hereditary factors, nervous system dysfunctions, bone metabolism impairment, metabolic pathways, and biomechanics [9,10]. The diagnosis of AIS is based on clinical observation and is confirmed through radiological imaging. Early diagnosis increases the chance of successful conventional treatment and reduces the risk of surgical intervention. Identification of metabolic dysfunctions accompanying the AIS pathophysiology may lead to its early recognition and thus and more effective treatment. There is no clear evidence to link AIS to biochemical disturbances, although extensive studies in this field are being conducted. However, some observations have been made. Metabolomic investigations have revealed several differences in the biochemical profile between AIS patients and a healthy group. For example, it has been found that the levels of plasma oxoglutarate, arginine, N-acetylaspartate, and citrate were significantly increased, whereas the levels of fumarate, glutamate, L-glutamic acid, malate, and N-acetylglutamate were reduced in AIS patients [11]. Furthermore, proteome analysis has indicated that some proteins are under or overexpressed in AIS patients [12,13], e.g., fibronectin, calmodulin, and fibrinogen concentrations were significantly higher and positively correlated with the Cobb angle [14]. Disturbance in the lipid profile has also been suggested as a marker of AIS [15]. In addition, the literature data indicate that abnormal levels of many hormones, including estrogen, melatonin, growth hormone, leptin, adiponectin, calmodulin, and ghrelin, may be related to the development and progression of AIS [10,16,17,18,19,20]. Furthermore, recently published studies have highlighted body composition as an essential factor in the onset and development of AIS [21,22]. 

In general, AIS patients are characterized by reduced bone mineral density (BMD) and bone mineral content (BMC), which may suggest dysregulation of calcium–phosphate turnover [23,24,25]; however, the observations are contradictory [26,27]. Therefore, more specific factors responsible for bone metabolism are taken into account, e.g., bone resorption marker (TRAP5b), sclerostin, osteoprotegerin, osteocalcin, receptor activator for nuclear factor κ B ligand (RANKL), and alkaline phosphatase [24,28,29,30,31]. Newly discovered bone turnover markers, such as C-terminal telopeptide of type I collagen (CTX) and N-terminal propeptide of type I procollagen (PINP), Klotho protein, and fibroblast growth factor-23 (FGF-23), are also being considered [32,33]. However, the data regarding metabolic disturbances in AIS are still insufficient, and the available information is often contradictory or ambiguous. Therefore, further investigations are needed, and our work aligns with this trend and aims to provide additional data to deepen our understanding of this issue. 

## 2. Results

### 2.1. Characteriscic of the Participants

Thirty-six age-matched female patients (AIS group) and eighteen healthy girls (control) were recruited for the study. A total of 36.1% of the AIS patients had a single spinal curvature, 55.6% had a double spinal curvature, and 8.3% had a triple spinal curvature. The participants’ demographic and basic clinical data are summarized in Table 1 and Table 2. All girls were in the postmenarcheal period, and there were no statistically significant differences in their age. However, the AIS group was characterized by lower body weight than the control (*p* < 0.015). 

Basic physiological parameters were assessed in all participants, including the level of natrium, potassium, creatinine, urea, and C-reactive protein (CRP) to exclude persons with impaired kidney function and acute inflammatory state (Table 3).

The values obtained for each patient were within the acceptable range, and no significant differences in the levels of the investigated factors were observed between the control and AIS patients, with the exception of CRP, which was higher in the control but did not exceed the physiological level (norm: 0.0–0.5 mg/dL).

### 2.2. Calcium and Phosphate Metabolism 

**Figure 1:**
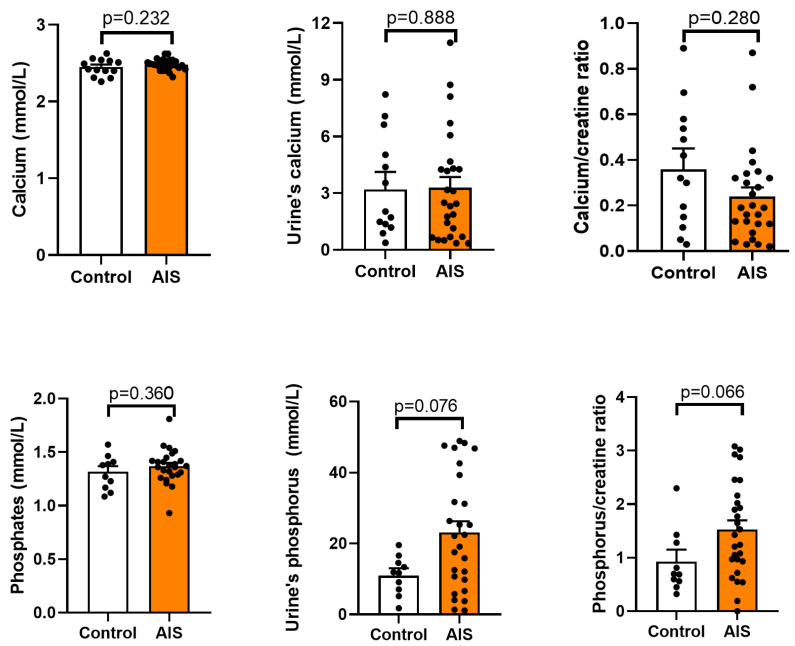
Level of calcium, and phosphates in the blood and urine of the control and adolescent idiopathic scoliosis female patients (AIS). Statistical differences between the control and AIS groups were measured using the *t*-test or Mann–Whitney U test, depending on the data distribution.

The concentration of phosphates and calcium in the blood and in the urine was assessed in the AIS and control groups (Figure 1). No statistically significant differences were found between the groups with *p* values for the AIS group vs. the control of 0.232, 0.888, 0.360, and 0.076 for calcium, urine calcium, phosphates, and urine phosphates, respectively. The obtained values were within physiological ranges. 

### 2.3. Regulators of Mineral Metabolism

**Figure 2:**
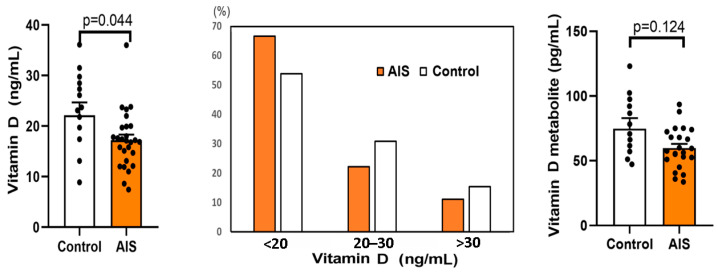
Level of vitamin D, its percentage distribution, and 1,25-dihydroxyvitamin D in blood samples from the control and adolescent idiopathic scoliosis female patients (AIS). Statistical differences between the control and AIS groups were measured using the *t*-test or Mann–Whitney U test, depending on the data distribution.

**Figure 3:**
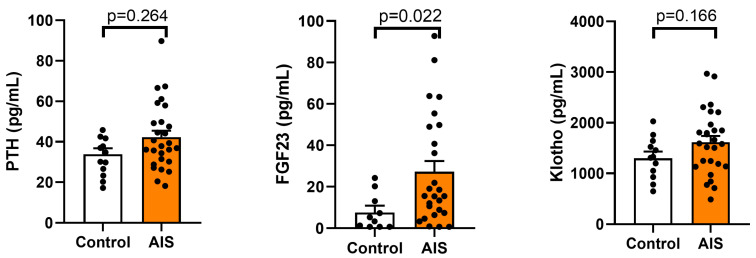
Level of parathormone (PTH), fibroblast growth factor 23 (FGF23), and Klotho protein in the serum of the control and adolescent idiopathic scoliosis female patients (AIS). Statistical differences between the control and AIS groups were measured using the *t*-test or Mann–Whitney U test, depending on the data distribution.

Vitamin D, parathormone (PTH), fibroblast growth factor 23 (FGF-23), and its cofactor Klotho protein are basic calcium and phosphate homoeostasis regulators. The concentrations of these factors in the serum of the AIS and control groups are shown in Figure 2 and Figure 3. 

The mean levels of serum 25-OH-vitamin D3 were 18.49 ± 6.81 ng/mL and 20.68 ± 7.16 ng/mL in the AIS and control groups, respectively. The difference between the AIS and control groups was statistically significant (*p* = 0.044). In terms of the distribution, 66.7% of the AIS patients had a deficiency of vitamin D (below 20 ng/mL) vs. 53.8% of the control, 22.2% of the AIS patients had insufficiency (between 20 and 30 ng/mL) vs. 30.8% of the control, and only 4 patients (11.1%) had a sufficient vitamin D level (above 30 ng/mL) vs. 15.4% of the control. No statistically significant difference was found for 1,25-dihydroxyvitamin D, i.e., an active metabolite of vitamin D formed in the liver.

The differences in the parathormone (PTH) level between the AIS group (mean: 40.20 ± 16.52 pg/mL) and the control group (33.55 ± 1.81 pg/mL) and in the Klotho protein content (1473.3 ± 631.5 pg/mL vs. 1150.8 ± 453.1 pg/mL) showed no statistical significance (*p* = 0.264 and 0.166, respectively). 

In turn, a significant increase in the level of fibroblast growth factor 23 (FGF-23) was observed in the AIS group (approx. 3.25-fold compared to the control). The mean values were 29.29 ± 30.8 pg/mL in AIS vs. 6.83 ± 8.2 pg/mL in the control group (*p* = 0.022) (Figure 3).

### 2.4. Biomarkers of Osteolysis and Osteogenesis

**Figure 4:**
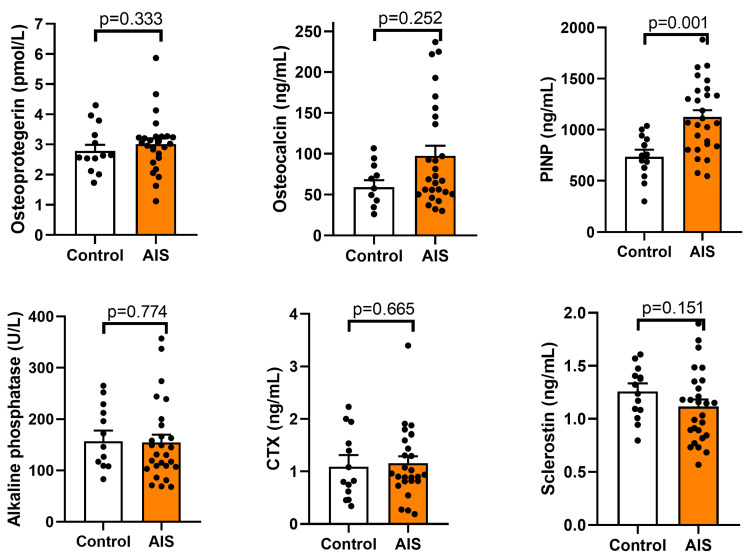
Level of selected biomarkers of osteolysis and osteogenesis in serum samples from the control and adolescent idiopathic scoliosis female patients (AIS). Statistical differences between the control and AIS groups were measured using the *t*-test or Mann–Whitney U test, depending on the data distribution.

Bone remodeling was evaluated using markers involved in bone formation, including osteocalcin, PINP, and osteoprotegerin and markers related to bone resorption: CTX, sclerostin, and alkaline phosphatase. The results are presented in Figure 4. The differences in osteoprotegerin, CTX, sclerostin, and alkaline phosphatase showed no statistical significance with *p* = 0.333, *p* = 0.665, *p* = 0.151, and *p* = 0.774, respectively. An elevated level of osteocalcin in the AIS group vs. the control group was observed with mean values of 97.6 ± 64.8 ng/mL and 52.9 ± 22 ng/mL, respectively; however, the difference did not exceed the significance level (*p* = 0.252). In turn, PINP was significantly higher in the AIS patients with the mean content 1104.2 ± 345.6 ng/mL vs. 808.2 ± 250.1 ng/mL in the control (*p* = 0.001).

### 2.5. Comparison between Different Age Groups

**Figure 5:**
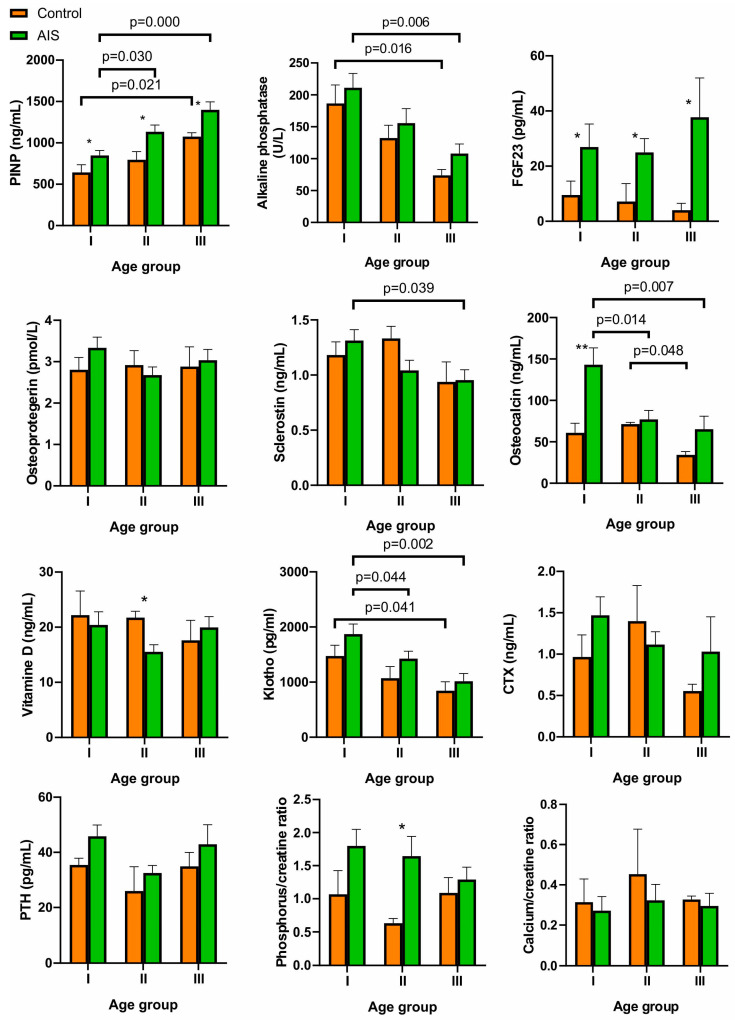
Comparison of the levels of the investigated factors in various age groups. Data are presented as mean ± SE (standard error). Statistical differences between the control and AIS groups were measured using the *t*-test or Mann–Whitney U test, depending on the data distribution: * *p* < 0.05, ** *p* < 0.01. The differences between the age groups were estimated using Tukey’s HSD test for normally distributed data or the Kruskal–Wallis H test for non-normally distributed variables, as determined by the Shapiro–Wilk test. *N* = 13, *n* = 13, and *n* = 10 in group I, II, and III, respectively.

The participants of the study were divided into different age groups based on the Risser sign, a method used to assess skeletal maturity. The analysis of the data from individual classes of the AIS patients showed that PINP increased with increasing skeletal maturity, and this tendency was similar in the control and study groups (Figure 5). The differences in PINP between the AIS group and the control were statistically significant in all age groups. In turn, alkaline phosphatase, sclerostin, and Klotho protein gradually decreased, but there were no differences between the AIS patients and the control. Furthermore, osteocalcin was significantly higher in the AIS I group compared to the control; however, a decreasing trend was observed in the AIS III group in both the AIS and control groups. The content of FGF23 was independent of skeletal maturity; however, in all age groups, the differences between the AIS and control groups reached a significance level. No differences were observed in the other tested factors, both between the control and AIS groups and between the different age groups.

### 2.6. Principal Component Analysis (PCA) 

In this paper, given the extensive exploration of numerous variables concerning AIS, principal component analysis (PCA) is utilized as a valuable tool for investigating multivariate data. It enabled the visualization of relationships among different groups of individuals (AIS, control, various age levels) within a two-dimensional variable space.

**Figure 6:**
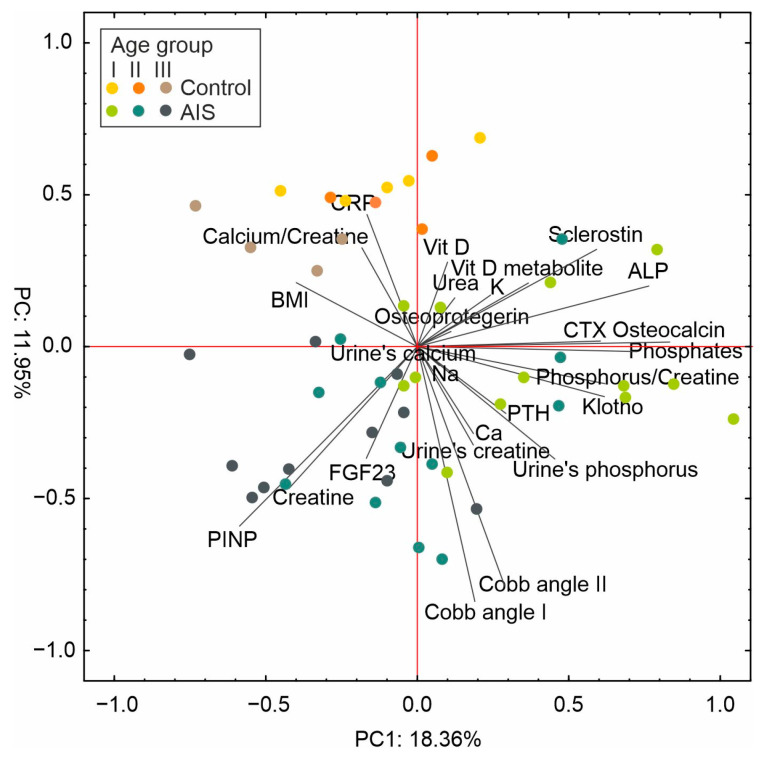
Scaled scatter plot of the principal component analysis of variables. The length of the lines represents the correlation between the original data and the factor axes.

Principal component analysis (PCA) revealed that the first two components explained more than 30% of the total variability (Figure 6). Specifically, PC1 accounted for approximately 18% of the total variability, while PC2 explained around 12%. PC1 showed positive correlations with phosphates, osteocalcin, phosphorus/creatine ratio, klotho, CTX, ALP, and sclerostin, whereas PINP, creatine, and BMI negatively influenced this factor. On the other hand, PC2 exhibited mainly negative correlations with the Cobb angle and slightly positive associations with CRP, calcium/creatine ratio, and vitamin D levels.

PCA of the selected variables clearly grouped the control and AIS individuals. In general, the second factor was responsible for the separation of the AIS samples, most of which were located below the X-axis, in contrast to the control individuals. Additionally, the samples showed a slight grouping based on the age stage. The first component facilitated the separation of the older patients (age group III), situated on the left side of the Y-axis, from the youngest patients, located on the right side of the Y-axis (age group I).

## 3. Discussion

Musculoskeletal disorders, which encompass a range of conditions affecting the bones, muscles, joints, and connective tissues, are a significant problem affecting a large portion of the population [34]. Among them, scoliosis occurring during adolescence requires great attention as it disrupts the development of a young body and leads to serious health consequences, such as back pain or respiratory issues, and has a major impact on quality of life. The imbalance in calcium and phosphorus levels, which are essential components for bone formation and remodeling, along with the disruption of osteolysis and osteogenesis, can be one of the causes contributing to the development of various musculoskeletal disorders [35,36]. Therefore, in our work, certain factors related to bone metabolism, including biomarkers involved in bone formation and bone resorption, reflecting the dynamic processes of bone remodeling, were considered potential contributors to the pathogenesis of adolescent idiopathic scoliosis (AIS).

To unify the group and avoid confusion related to gender and the maturation process, only girls in the postmenarcheal period participated in our study. There was no difference in age between the AIS and control groups; however, the AIS group was characterized by a lower body weight, which is typical for patients with scoliosis. [32].

Calcium and phosphate metabolism is regulated by numerous factors, with the greatest significance of the PTH/1,25(OH)2D and FGF-23/Klotho axis [37]. PTH decreases renal calcium excretion, inhibits phosphate reabsorption, and stimulates 1,25(OH)2D synthesis. It also acts directly on bone, resulting in increased calcium and phosphate efflux and the resorption of mineralized bone. In turn, 1,25(OH)2D plays a role in regulating the absorption of calcium ions from the digestive system and connects both regulatory axes [37,38]. Fibroblast growth factor 23 (FGF-23) and its cofactor, Klotho protein, which play a role in phosphate control, are relatively less widely studied markers of bone turnover. The production of FGF23 by osteoblasts and osteocytes is primarily stimulated by 1,25(OH)2D and phosphate loading. However, PTH, calcium, and sclerostin also participate in regulating FGF23 [39,40]. Klotho increases the affinity of fibroblast growth factors (FGFs) for their respective receptors. In general, all these factors are responsible for maintaining phosphate and calcium homeostasis in the body.

In present study, we did not observe any differences in the calcium and phosphate levels between the AIS and control groups. The values determined in each participant included in the study were within the normal ranges (0.8–1.44 mmol/L for phosphate and 2.25–2.75 nmol/L for calcium), which aligns with some of the literature data [41,42]. Furthermore, similarly to the majority of reports [27,41,42], the PTH levels in all participants were within the reference limit (15–65 pg/mL). In turn, the concentration of vitamin D was below the reference range in most participants, and a statistically lower level of vitamin D was observed in the AIS group compared to the control (*p* = 0.044). No statistically significant difference was found in 1,25(OH)2D, i.e., the active metabolite of vitamin D, which sometimes serves as an indicator of vitamin D status. However, the metabolite is unstable, and it has been shown that it does not reflect dietary vitamin D intake [43]. Interestingly, a 3.25-fold higher level of FGF-23 was observed in AIS compared to the control group, which could potentially explain the elevated urine secretion of phosphate observed in some AIS patients. However, it should be noted that the differences between the AIS and control groups did not reach a statistically significant level.

Although our findings agree with much of the literature data, there are also some contradictory observations. For example, Goździalska et al., who compared calcium and phosphate levels in pre- and postmenarcheal girls with AIS and a control group, reported that calcium levels were lower in the AIS group compared to the control. These differences were particularly evident in postmenarcheal girls. On the other hand, phosphorus levels in the postmenarcheal AIS group were higher than in the postmenarcheal control group [27]. Furthermore, a decrease in PTH was observed in the postmenarcheal AIS girls [27]. The significance of the vitamin D level in the progression of AIS was considered in numerous studies as well [44,45]; however, the results are still inconclusive. Most researchers suggest reduced vitamin D levels in AIS patients [27,41,46]. Conversely, other authors found no association [47,48]. Based on a systematic review and meta-analysis of available data, Zhu et al. found that the AIS group had a lower vitamin D level than the control and suggested that its deficiency may be involved in the pathogenesis of AIS [26]. Calcium and calcitonin levels were also moderately lower in AIS patients; however, there was no difference in phosphorus and parathyroid hormone level between the AIS and control groups. 

Currently, there are limited reports in the literature on Klotho protein and FGF-23 levels in adolescent idiopathic scoliosis. Brzęczek et al. found that Klotho protein levels in children with scoliosis were significantly lower than in the control group. Additionally, scoliotic patients exhibited a notable trend towards higher FGF23 levels compared to the control group [33]. Furthermore, similar FGF-23 disturbance has been observed in other types of musculoskeletal disorders [49,50]. This supports our findings regarding FGF23 and suggests that the FGF23-Klotho axis may be involved in AIS pathophysiology; therefore, it is worth continuing this research with a larger group of patients.

Bone turnover is also regulated by many other molecules involved in bone formation and bone resorption. For example, osteocalcin regulates bone quality by aligning biological apatite (BAp) parallel to the collagen fibrils and may interact with collagen and determine the BAp growth direction [51]. Osteoprotegerin (OPG) can bind to RANKL and therefore inhibit RANK/RANKL signaling, suppressing the maturation and activation of preosteoclasts. As a result, it protects against excessive bone resorption. In turn, sclerostin, expressed by mature osteocytes and articular chondrocytes, is an endogenous inhibitor of the Wnt signaling pathway involved in regulating bone formation and regeneration. Sclerostin binds to its receptors on the cell surface of osteoblasts, initiating intracellular signaling with the ultimate effect of inhibiting bone formation [40,52]. Alkaline phosphatase increases phosphate local rates, facilitates mineralization, and decreases extracellular pyrophosphate, an inhibitor of mineral formation [53]. In our study, none of the aforementioned factors were found to be disturbed in the AIS patients compared to the healthy control group. 

Serum PINP and CTX are additional biochemical markers used to assess the process of bone turnover. PINP, i.e., a by-product of collagen synthesis, is associated with bone formation, and the activity of osteoblast lineage cells and CTX, a product of degradation type I collagen, reflects osteoclast activities and bone resorption. In our study, a significantly increased level of PINP was noted in the AIS group compared to the control; however, no statistically significant difference in CTX was observed. A similar observation was reported by Zhang et al. [32] These results indicate more active bone turnover in AIS patients than in healthy controls.

We are aware of several limitations of our study, including the small sample size of patients with AIS and healthy participants and the fact that this was a single-center study. Furthermore, male adolescents with AIS were not included in the study. As the prevalence of AIS in adolescent females is higher than in males, it was not possible to obtain a sufficiently large male group for statistical analysis. To overcome these limitations, prospective multicenter studies should be planned for the future to include a larger group of patients with AIS in the investigation.

## 4. Materials and Methods

### 4.1. Selection of Participants

The investigation involved children (females) aged 11 to 18 years old who were patients at the Pediatric Orthopedic Clinic of the Medical University of Lublin in 2016–2018. Healthy volunteers were the control group. The recruitment of participants for the control group was conducted among female adolescents who reported to the orthopedic clinic. The primary inclusion criterion was the absence of orthopedic issues based on clinical observation. The other criteria for the control group included being aged from 11 to 18 years, having no history of fractures in the last 12 months, and not having any chronic diseases. Additionally, their parents had to consent to their participation in the study. From the initial group of thirty-four qualified individuals, four individuals were finally excluded because they were in the premenarcheal period, and parental consent was not obtained in the case of twelve persons.

The study group was recruited from AIS patients who underwent clinical and radiological examinations to confirm scoliosis. The inclusion criterion was the presence of spinal deformity with Cobb angles above 40°, indicating progressive scoliosis. The exclusion criteria included chronic diseases (e.g., liver or endocrine dysfunction, diabetes, disturbance of calcium–phosphorous metabolism), fractures during the one-year period prior to the beginning of the study, treatment based on antiepileptic drugs and steroids, scoliosis with known etiology, muscular dystrophy, and neuromuscular disorders. Out of the initial thirty-seven individuals qualified for the study group, two females were excluded as they were in the premenarcheal period. 

The participants of the study did not have any limitations in physical activity, and they actively participated in physical education classes. Additionally, the children with AIS had approximately 30 min of exercise as part of individual physiotherapy. None of the participants engaged in sports professionally. One person from the control group participated in additional sports activities once a week for 2 h. The majority of the participants were on a normal diet without any restrictions. One person from the control group and two people from the study group were on a vegetarian diet.

The study was approved by the Bioethics Committee of the Medical University of Lublin (consent no. KE-0254/105/2015; KE-0254/74/2017). Written informed consent was obtained from all participants and their parents.

### 4.2. Study Design

All participants of the study underwent clinical observation, and a detailed interview regarding nutrition, physical activity, medications, and medical conditions was conducted. Additionally, they were subjected to X-ray examination to assess the type and degree of deformity (AIS group) and skeletal maturity (the control and AIS group). In the morning, blood and urine samples were gathered from the participants for biochemical analyses.

#### 4.2.1. X-ray Examination

The Cobb angle was measured using digital radiography based on anteroposterior X-ray according to a procedure published previously [54]. Skeletal maturity of the patients was based on the Risser sign. The test is based on the evaluation of iliac crest apophysis. The sign is graded from 0 to 5: grade 0 indicates no ossification; grade 1 indicates ossification within the first quarter (25%) of the crest; grade 2 indicates ossification extending into the second quarter (25–50%) of the crest; grade 3 indicates ossification into the third quarter (50–75%) of the crest; grade 4 indicates ossification into the fourth quarter (>75%) to completion of the apophyseal line excursion; and grade 5 indicates complete ossification of the iliac crest [55].

#### 4.2.2. Biochemistry Assay

Blood samples were collected in tubes with EDTA (1 mg/mL) (Sarstedt Monovette EDTA KE, Nümbrecht, Germany) and immediately centrifuged at 1500× *g* for 10 min at 4 °C (Eppendorf centrifuge). The supernatant (plasma) was separated and stored at −20 °C until measurement. Urine was collected in sterile containers.

Levels of serological markers were measured using an enzyme-linked immunosorbent assay kit (ELISA). Procollagen I N-Terminal Propeptide (PINP) kit was from CLOUD-CLONE CORP. (Katy, TX, USA), Serum CrosLaps (CTX-1) Elisa, and N-MID Osteocalcin Elisa were from IDS (Boldon, UK), Human Sclerostin HS EIA kit was from TECOmedical Group (Lučko-Zagreb, Croatia), Human Osteoprotegerin Elisa was from Bio-Vendor (Brno, Czech Republic), Human FGF-23 Elisa Kit was from Immutopics Inc (Athens, OH, USA) and Human Soluble a-Klotho Assay Kit was from Tecan (Männedorf, Switzerland). The procedures were performed according to the manufacturer’s instruction.

### 4.3. Statistical Analysis 

The data are expressed as mean values ± SE. The normality of continuous data (*p* < 0.05) was evaluated using the Shapiro–Wilk test. For variables that were normally distributed, the *t*-test or ANOVA followed by Tukey’s post-hoc test was conducted (*p* < 0.05). In the case of non-normally distributed variables, the Mann–Whitney U test or Kruskal–Wallis H test was used (*p* < 0.05). The data were subjected to principal component analysis (PCA). Statistical analysis was performed using Statistica ver. 13.3 software (TIBCO Sofware Inc., Palo Alto, CA, USA).

## 5. Conclusions

Our study demonstrated that the phosphate–calcium balance and the PTH level appear to be normal in AIS patients. However, we observed decreased serum levels of 25-OH-D and increased levels of FGF23 and PINP in the girls with AIS compared to the healthy girls. A deficiency of vitamin D can lead to a decrease in the absorption of calcium. In turn, an increased level of FGF23 can result in greater urinary excretion of phosphate and reduced phosphate reabsorption. Disruptions in these factors can contribute to decreased bone mineralization. Higher levels of P1NP indicates increased bone formation and turnover.

Our study may suggest the involvement of these factors in AIS progression, although further studies are required to elucidate the underlying mechanisms of idiopathic scoliosis.

## Figures and Tables

**Table 1:** Demographic and basic clinical data of participants.

Parameter	Control (*n* = 18)	AIS Group (*n* = 36)	*p*
Age (year)	14.60 ± 2.10	14.64 ± 1.81	0.781
BMI (kg/m^3^)	23.38 ± 3.87	20.50 ± 3.88	0.019
Cobb angle I (°)	-	52.78 ± 16.79	-
Cobb angle II (°)	-	50.82 ± 17.41	-
Cobb angle III (°)	-	63.0 ± 7.21	-

**Table 2:** Type of curvature according to Lenke classification.

Description	Type	No of Patients
Main thoracic (MT)	1	10
Double thoracic (DT)	2	3
Double major (DM)	3	9
Triple major (TM)	4	3
Thoracolumbar/lumbar (TL/L)	5	4
Thoracolumbar/lumbar-main thoracic (TL/L-MT)	6	3

**Table 3:** Basic physiological parameters.

Parameter	Control (*n* = 18)	AIS Group (*n* = 36)	*p*
Natrium (mmol/L)	138.71 ± 1.82	138.37 ± 1.90	0.555
Potassium (mmol/L)	4.278 ± 0.269	4.273 ± 0.278	0.602
Creatinine (mg/dL)	0.602 ± 0.081	0.683 ± 0.098	0.061
Urine creatinine (mg/dL)	139.99 ± 68.29	166.89 ± 93.93	0.571
Urea (mg/dL)	22.91 ± 6.71	22.22 ± 5.51	1.000
CRP (mg/dL)	0.095 ± 0.046	0.067 ± 0.028	0.009

## Data Availability

The data presented in this study are available on request from the corresponding author.
